# Barriers and facilitators to adherence for antiretroviral therapy: the perspectives of patients from a wellness center in the Mpumalanga Province, South Africa

**DOI:** 10.4314/ahs.v22i3.49

**Published:** 2022-09

**Authors:** Portia T Simelane, Maswati S Simelane, Acheampong Y Amoateng

**Affiliations:** 1 Department of Demography and Population Studies, Northwest University, Mafikeng, Northwest Province, South Africa; 2 Department of Statistics and Demography, Faculty of Social Sciences, University of Eswatini, Eswatini; 3 School of Research Studies, Northwest University, Mafikeng Campus, Northwest Province, South Africa

**Keywords:** ART, Barriers, Facilitators, HIV/AIDS, South Africa

## Abstract

**Introduction:**

Patients' non-adherence to antiretroviral treatment remains a public health concern in many developing countries, especially in South Africa.

**Objectives:**

The objective of the study was to explore the barriers and facilitators of patients' ART adherence in one health care facility in Mpumalanga Province, South Africa.

**Methods:**

A qualitative, exploratory, and descriptive design was employed to collect data using a semi-structured interview guide through individual in-depth interviews among twenty (20) purposively selected patients. The thematic analysis approach was used to generate themes from the data.

**Results:**

A majority of the participants were female (n=12, 60%), married (n=13, 65%), and employed (n=12, 60%). Barriers to ART adherence include insufficient medical staff at the health center and waiting time being too long. Facilitators included service providers' positive attitude, clear instructions for taking medication, benefits of adhering to ART, and dangers of defaulting treatment.

**Conclusion:**

Barriers and facilitators for adherence included several factors related to the health system, health care workers, and the patients. Achievement of optimal adherence to ART requires the commitment of both patients and providers.

## Introduction

The benefits of early initiation of antiretroviral therapy (ART) has long been recognised 1–3. ART decreases the transmission of HIV and the progression of Acquired Immune Deficiency Syndrome (AIDS) and improves the quality of life 4, 5. In 2017, in South Africa approximately 7.9 million people were living with HIV (PLWHIV), and the overall prevalence of HIV among adults aged 15 and older was 18.8% 6, 7. Of all the PLWHIV in South Africa, 70.7% were on ART, and 87% had achieved viral suppression in 2017 7. As a result of ART's scale-up in South Africa, there was a drastic reduction in AIDS-related deaths from 267,417 in 2007 to 126 805 in 2019 8.

Adherence to ART is essential to realise the effectiveness of the medication and positive health outcomes. To optimize ART, 95% adherence to unboosted protease inhibitors (PIs) should be achieved while at least 80% for adherence to boosted PIs 9, 10. Poor adherence or non-adherence to ART results in poor health outcomes, such as HIV drug resistance (HIVDR) mutations and mortality^11^–^13^. Evidence suggests that in Africa the prevalence of HIVDR has been fuelled by poor ART adherence among other factors 13, 14.

Several studies in South Africa have indicated non-adherence to be a problem 15–17. ART non-adherence has been a dynamic behaviour related to interpersonal level factors, structural factors, and healthcare workers (HCWs) failure to explain the importance of the medication adequately and poor interaction with patients 18, 19. A systematic literature review conducted in Sub-Saharan Africa (SSA), Asia and Europe found that lack of knowledge on the benefits of ART treatment and adverse effects of the medication, patients' age, education level and place of residence were some of the factors that affected ART adherence^20^–^22^. Interpersonal factors are related to the patients' partner, the patient family, the use of health facilities, and HCW attitudes towards patients 23–25. At the community level, stigma was reported by several studies to have a negative effect on ART adherence 26–28. Among the structural issues, access to health care services and the use of health care services were found to influence ART adherence^11^, ^19^. Despite the available evidence in the literature, more studies are required to unearth the multiple factors contributing to the patients' poor adherence to ART. Therefore, we aimed to gain a deeper understanding of the barriers and facilitators of ART adherence from the patients' perspective.

## Methods

### Study design

This study was a qualitative component of a mixed-method study^29^. The qualitative component of the study adopted an exploratory, descriptive design conducted between June 2017 to August 2017 at a Health Center in Mpumalanga, South Africa. The exploratory research methodology is suitable for this study. An exploratory study is designed to explain how a phenomena manifested and important to uncover the full nature of an inadequately understood phenomenon^14^. Utilization of the qualitative approach in the mixed methods was appropriate since we aimed to capture a deeper understanding and perspectives of the patients on the facilitators and barriers of ART adherence.

### Sampling method and procedure

The wellness center was accredited in 2005 as part of the hospital in Mpumalanga. Since then, it has provided a comprehensive package of HIV and AIDS, and TB, including pre and post counselling and continued education on patients' adherence. It is situated under the Gert Sibande district municipality with a population of 943,137 in the Mpumalanga Province. It is situated in the eastern boundary of Mpumalanga, boarded by Eswatini in the East, KwaZulu-Natal in the South, Free State in the West, and Gauteng North.

There were 7,773 patients enrolled in ART between 2010 and 2014 recorded in the facility's electronic database. However, from the mixed methods study, 777 patients were enrolled using probabilistic methods of which 291 were defaulters and 486 were none defaulters^29^. For the qualitative section of the mixed-method study, purposive sampling was used to recruit 20 participants (10 defaulters and 10 non-defaulters) aged 15–49 years from the 777 patients. Purposive sampling was the preferred approach since it allowed for the selection of participants that were deemed to provide unique and rich information about the phenomena 30. It was possible to identify participants that were eligible for the study since their HIV records and status of ART were recorded in the facility's database. The patients' routine appointments for ART refill were scheduled by the HCWs. The HCWs introduced the researcher to the potential participants. Only patients who consented to be part of the study and were defaulters meaning they missed some medical appointment and those that were non-defaulters were enrolled in the study. A face to face interview was used to collect data from the twenty participants about their perspectives on ART adherence.

### Data Collection

We used a semi-structured interview guide and a voice recorder to collect qualitative data from the participants who enrolled in the study. The semi-structured interview guide was in isiZulu and was translated to English before the interviews and participants were interviewed by the researcher. All participants preferred to be interviewed in IsiZulu.

The interview guide focused on HIV/AIDS, barriers and facilitators to ART adherence, benefits of adhering to ART, physiological body changes on the patients resulting from ART uptake, and service quality at the wellness center. After posing each question, the researcher used probes to gain more rich information of the participants. Data saturation was deemed to have been reached when the data collected represented the construct we investigated and no new codes were imminent from the interviews^31^. Each participant was interviewed for about 40 minutes and data was audio-recorded and transcribed.

### Data analysis

The recordings of the interviews from the voice recorder were transcribed verbatim into the analysis software NVIVO 8^32^. Two researchers (PS and AA) first read the transcripts to familiarize themselves with the data and to construct preliminary codebooks. A systematic, comparative and descriptive content analysis was performed by re-reading each transcript and applying codes to thematically identify similar passages of texts. To resolve disagreements between the researchers, the researchers would discuss the interpretation of the codes and revisits the codes transcripts in order to reach consensus on the meaning of the transcripts. Codes were applied to the themes that emerged from the transcripts. The text was then aggregated by each code and extracted into matrices to identify recurring issues and differences in the narratives of the data. Based on the qualitative analysis, five themes emerged: (i) perspectives on service provider staffing, (ii) waiting time at the clinic, (iii) perspectives on the attitude of service providers, (iv) perspectives on clear instructions for taking medication, (v) perspectives on the benefits of adhering to ART and (vi) perspectives on the dangers of not adhering to ART.

### Study trustworthiness

To ensure trustworthiness, conformability was ensured by ensuring having each of the researchers independently coding the data in order to ensure consistency among the identified themes. Peer review of the codes and themes was done by other experienced qualitative researchers to ensure the credibility of the themes. Detailed field notes were used during the transcription and analysis of data for audit checks and verification. The use of a semi-structured data collection tool allowed the respondents to share their perspectives freely on the subject and probes were used to solicit more rich information.

### Ethical consideration

The study materials were reviewed and approved by the Northwest University, Mafikeng, South Africa ethics committee, and the Mpumalanga National Health Research Committee. Written administrative approval to conduct the study was obtained from the Wellness center in Mpumalanga from management. Written informed consent was obtained from all participants that took part in the study and were archived in a lockable cabinet that was accessed by other study researchers along with the principal investigator. Participants' names were not required during the interviews to protect their anonymity. The names of the participants were not required at any point of data collection process in order to protect their identity and during data analysis, transcripts that had potential identification of the participants were de-identified to protect the identities of the participants. As per the guidance of the ethics committees, the data will be destroyed after three years.

## Results

A total of 20 participants were interviewed in the study before saturation was reached. Half (50%) of the participants were defaulters of the ART medication, and most (60%) were females. The mean age of the participants was 37 (SD: 11.5) years and slightly over half (55%) were residents in urban areas, with 40% being unemployed (Table 1).

**Table 1:** Participants' characteristics

Patient characteristics	n (%)
**Age** (mean and standard deviation)	37; SD. 11.5
**Sex**	
Male	8 (40.0)
Female	12 (60.0)
**Marital status**	
Married	13 (65.0)
Not married	7 (35.0)
**Employment status**	
Employed	12 (60.0)
Unemployed	8 (40.0)
**Residence**	
Rural	9 (45.0)
Urban	11 (55.0)
**ART Defaulter**	
Yes	10 (50.0)
No	10 (50.0)

### Perceived barriers to ART adherence Inadequate Staff

In general, all (20) of the respondents considered staffing to be inadequate. According to the patients, insufficient medical personnel compromised their treatment adherence, as little attention was afforded to them. A majority (18) of the respondents cited that the nurses and the doctor were overwhelmed, with insufficient time to engage and assess patients' concerns about ART adherence. Only one doctor and three to four nurses were expected to attend more than 150 patients per day. According to the respondents, the number of medical personnel was inadequate to engage in dialogue with patients due to insufficient time. A 29 years old woman who was a defaulter shared her view on the problem of insufficient medical staff in the facility;

“*We need more nurses and doctors in this facility. The government should add more health workers, currently; we have only one doctor and four nurses a day. This then makes us wait many hours before we get attended to. Imagine one doctor having to attend to more than 150 patients a day! If one arrives in the morning, the waiting time is much shorter and bearable. Still, suppose you arrive after 1:00 pm since there would be more people, including those who would have started at work in the morning like myself before coming to the hospital to collect their ARVs. In that case, the waiting time then becomes very long*” (Defaulter, female, 29 years old, employed, from a rural area).

Another participant shared his dissatisfaction with the limited staff: “*I am not happy with the current staffing at the wellness center. Since I have to start at work before coming to the wellness center to collect my ARVs, I always arrive here around 1:00 pm. On arrival, the health workers would have gone for lunch, and we would patiently wait to be attended. The health care providers are very limited in numbers with only one doctor and around 4 or 5 nurses at most for more than 150 patients daily*”(Defaulter, male, 28 years, employed, from an urban area)

### Waiting time

The participants expressed dissatisfaction with the long waiting time before being attended to by the medical staff. They expressed that it was better before lunch when the medical staff was still energetic, but the service was poor after lunch. This is what one of the defaulting patients had to say;

“*Given that I stay in the farms and transport from there to the hospital is very scarce, I always arrive in the hospital after lunch. Due to the long queues, one end up finishing after 5 pm. This becomes a challenge as some of us are coming very far, so we end up even missing our transport back home*”. (Defaulter, female, 29 years, employed, rural residence)

Another patient expressed her views as follows: “*Do you think I will be in a position to lose a whole day's pay from my salary because I have to come and wait here the whole day? Never. Some of us have to beg our employers to give us a few hours to come and collect our medication. But when you arrive here, you find yourself sitting here for the whole day before you are attended to, yet you still have to go back to work, and our employers say no-work, no-pay*”. (Non-defaulter, male, 41 years, employed, from an urban area)

It was noted that some of the participants, especially those who arrived in the morning hours, were pleased with the facility's service and waiting time. This is what one participant had to say. “*I personally have no problems with the staffing of the health workers and waiting time, especially because I always collect my medication early in the morning. But I once waited for my friend, who arrived after lunch, to collect her ARVs. I should say that I did see a great need to have more health workers added to assist here at the facility. My friend even confided to me that the reason why she once defaulted was because of the long waiting times when she had to come and collect her medication at the clinic*.” (Non-defaulter, female, 41 years, unemployed, from a rural area)

### Perceived facilitators to ART adherence

The participants mentioned several facilitators to ART adherence.

### The attitude of service providers

The majority (15) of the participants cited service providers' attitudes to be a factor for ART adherence. Generally, patients reported that service providers at the facility had a positive attitude towards them. They are caring and very helpful in supporting them to adhere to treatment. This is a narrative of one of the patients at the wellness center: “*The nurses and doctor treat us well. They have a very good attitude towards us; they always encourage us to take care of ourselves. They treat us well. Even today, I told them that I am rushing to work and they understood and quickly retrieved my file. The health workers respect our rights, too, so I am still taking treatment in this wellness center. Since I started taking treatment here, I have never changed the hospital because of how they treat us well. They actually made me stay. They even encourage us to take the treatment faithfully.*” (Non-defaulter, male, 46 years old, urban residence) However, some patients reported that they had experiences whereby service providers had a negative attitude towards them. The majority of these (5) were patients who had previously defaulted treatment. The patients cited the negative attitudes of HCWs as one of the main reasons they defaulted on their medication. This is a narrative from an unemployed woman, aged 30 years residing in an urban area:

“*They do not treat us well. This is a challenge for us because this place is where we should be running to get some help but the way they mistreat us! They do not even ask questions why you defaulted, my sister; there are challenges in life that we face out there. You get into circumstances whereby you cannot afford even transport money to come and collect medication. It is not like you do not want to come. When you arrive here, you get shouted at. This has resulted in other patients deciding rather die at home than to come here and get mistreated*.” (Defaulter, female, 30 years, unemployed, urban residence)

### Instructions for taking medication

One of the critical facilitators to ART medication was cited by the majority (18) of the patients. The majority of the patients were happy with the service providers' guidance on how to take the medication. A 41-year-old female, a non- defaulter, unemployed from the rural areas shared her view;

“*I was given clear instructions on how to take my medication. The lady explained everything to me; I don't know if the lady is still here. I asked her questions and she explained everything to me that since I am HIV positive now, I should take care of myself and take my ARVs daily as it is my life now. We were taught that we have to respect the time that we have chosen to take the medication because this is our life*”. (Non-defaulter, female, 41 years, unemployed, rural residence)

### Benefits of adhering to ART

This study took the initiative to investigate if patients understood the benefits and importance of generally adhering to ART. All the respondents of this study have indicated that it is very important to adhere to ART. Some of the narratives expressed by the patients are as follows: “*It is important to take your ARV's if you want to live, and if you want to die, then you can stop taking them. Taking treatment faithfully makes you healthy, and your body weight improves. I was so thin and I had lost a lot of weight before I started taking my treatment. Look at me right now! The pills have helped me a lot. My bodyweight has improved a lot, and I am healthy now*”. (Non-defaulter, Female, 29 years old, unemployed, rural residence).

### Dangers of defaulting treatment

Concerning the patients' perspectives on the dangers of defaulting treatment, all the patients' responses indicated a good understanding of the dangers of defaulting treatment. The following is the narrative from one respondent:

“*My sister, defaulting treatment is very, very, dangerous. When I look at my friends who became HIV positive earlier than me and defaulted treatment, all of them are dead now because after defaulting, they were too weak to come to the hospital to get help. But I was lucky because after defaulting, I returned to the hospital and got some help on time. I also asked for forgiveness from the health providers for not coming back to take my treatment. Then they allowed me to get back into treatment again*.” (Defaulter, Male, 30 years, Unemployed, Urban residence).

## Discussion

This study revealed several barriers and facilitators of ART adherence. Among the barriers to ART adherence we found; insufficient medical staff and long waiting times. Insufficient medical staff means a rapid increase in HCWs' workload, especially if the World Health Organisation (WHO) guidelines on the test and start 33 are to be implemented adequately. Having more staffing can address the concern for non-adherence to ART as patients can spend less time in facilities. Studies have shown the negative effects of insufficient medical staff on ART uptake and adherence^11^, ^34^. Increased workload for HCWs has been a significant problem, especially in Sub-Saharan Africa (SSA), where there is limited health systems infrastructure in the context of test and start ART^33^, ^35^.

The finding that long waiting time at ART clinics negatively impacts patients' adherence has been documented in other studies^36^, ^37^. As countries are geared towards achieving the 95-95-95 UNAIDS targets by 2030 38, they should also increase human resources to ensure staff have optimal workload and accommodate new clients' influx. The finding that the positive attitudes of HCWs are a major facilitator of ART adherence is in line with other studies^39^–^41^. Many of the patients reported that service providers at the facility had a positive attitude towards them. They were caring and very helpful in supporting them to adhere to treatment to ART. Patients satisfied with their healthcare provider are more likely to have high treatment success 41. This finding is consistent with the literature^42^,^43^. Given the limited time that patients are afforded by the HCWs due to understaffing, more patients could be discouraged to honour their medical appointments and adhering to ART. Government interventions of increasing the staff personnel at the wellness center could mitigate the problem of ART non-adherence.

Several studies have reported that lack or poor communication about the dangers of non-adherence remains a barrier to ART adherence 44–46. The study found that participants who understood the dangers of non-adherence reported being adherent to ART.

## Limitations

Limitations are inherent in the study. Participants were asked to recall their experiences on the barriers and facilitators, leading to recall bias. The study was conducted in only one health center in the Mpumalanga province. In contrast, several facilities across the country initiate and manage patients on ART, of which the patients in those facilities might have different perspectives on the phenomenon. The study's design does not permit the generalization of the results; instead, it should be interpreted with an in-depth nature to understand the phenomenon of ART adherence at the wellness center investigated.

## Conclusion

Barriers and facilitators for adherence included several factors related to the health system, health care workers, and the patients’. Achievement of optimal adherence to ART requires the commitment of both patients and providers. Information about the importance of ART adherence should be communicated from the initiation stage throughout the patient's lifetime. It is the government's responsibility through hospitals and clinics around the country to ensure that quality service is accessible and delivered to ART patients just in time to ensure lifetime adherence to ART. There is also a need to undertake further research to investigate barriers to ART across all wellness centers across the country to ensure that patients on ART do not default simply due to poor quality services.
